# Ultrasound-based classification of follicular thyroid Cancer using deep convolutional neural networks with transfer learning

**DOI:** 10.1038/s41598-025-05551-7

**Published:** 2025-07-01

**Authors:** Enock Adjei Agyekum, Zhang Yuzhi, Yu Fang, Doris Nti Agyekum, Xian Wang, Eliasu Issaka, CuiRong Li, Xiangjun Shen, Xiaoqin Qian, Xinping Wu

**Affiliations:** 1 Department of Ultrasound, Affiliated People’s Hospital of Jiangsu University, Zhenjiang, 212002 China; 2 School of Computer Science and Communication Engineering, Jiangsu University, Zhenjiang, Jiangsu Province China; 3 Department of Ultrasound, the Affiliated Hospital of Integrated Traditional Chinese and Western Medicine, Nanjing University of Chinese Medicine, Nanjing, China; 4 Department of Medical Laboratory Technology, University of Cape Coast, Cape Coast, Ghana; 5 Northern Jiangsu People’s Hospital Affiliated to Yangzhou University, Yangzhou, China; 6 Northern Jiangsu People’s Hospital, Yangzhou, Jiangsu Province China; 7 The Yangzhou Clinical Medical College of Xuzhou Medical University, Yangzhou, Jiangsu Province China; 8 College of Engineering, Birmingham City University, Birmingham, B4 7XG UK

**Keywords:** Follicular thyroid carcinoma, Follicular thyroid adenoma, Convolutional neural network, Artificial intelligence, Ultrasound, Thyroid cancer, Cancer

## Abstract

This study aimed to develop and validate convolutional neural network (CNN) models for distinguishing follicular thyroid carcinoma (FTC) from follicular thyroid adenoma (FTA). Additionally, this current study compared the performance of CNN models with the American College of Radiology Thyroid Imaging Reporting and Data System (ACR-TIRADS) and Chinese Thyroid Imaging Reporting and Data System (C-TIRADS) ultrasound-based malignancy risk stratification systems. A total of 327 eligible patients with FTC and FTA who underwent preoperative thyroid ultrasound examination were retrospectively enrolled between August 2017, and August 2024. Patients were randomly assigned to a training cohort (*n* = 263) and a test cohort (*n* = 64) in an 8:2 ratio using stratified sampling. Five CNN models, including VGG16, ResNet101, MobileNetV2, ResNet152, and ResNet50, pre-trained with ImageNet, were developed and tested to distinguish FTC from FTA. The CNN models exhibited good performance, yielding areas under the receiver operating characteristic curve (AUC) ranging from 0.64 to 0.77. The ResNet152 model demonstrated the highest AUC (0.77; 95% CI, 0.67–0.87) for distinguishing between FTC and FTA. Decision curve and calibration curve analyses demonstrated the models’ favorable clinical value and calibration. Furthermore, when comparing the performance of the developed models with that of the C-TIRADS and ACR-TIRADS systems, the models developed in this study demonstrated superior performance. This can potentially guide appropriate management of FTC in patients with follicular neoplasms.

**Supplementary Information:**

The online version contains supplementary material available at 10.1038/s41598-025-05551-7.

## Introduction

Thyroid tumors, particularly follicular thyroid carcinoma (FTC) and follicular thyroid adenoma (FTA) present significant clinical challenges due to their varying prognoses and treatment requirements. 10–15% of all thyroid tumors are FTC, making it the second most common kind of thyroid cancer after papillary thyroid carcinoma (PTC)^1^. Park et al. reported in 2009 that the thyroid imaging reporting and data system (TI-RADS) might be used to analyze the type of thyroid nodules, which received broad attention and acknowledgment^2^. However, some studies noted that the diagnostic performance of TI-RADS is typically favorable for PTC but less successful for FTC, emphasizing the necessity for other diagnostic methodologies when analyzing nodules thought to be FTC^3,4^. Patients diagnosed with FTC are often older than those with PTC, which is associated with a poorer prognosis. Several studies have demonstrated that older age is a significant risk factor for disease-specific mortality in FTC. Moreover, the mean age at diagnosis for FTC is higher than that for PTC, which presents challenges in confirming FTC ^5,6^.

Recent studies have focused on the differentiation between FTC and FTA using ultrasound (US) imaging, highlighting the challenges and potential diagnostic features^7,8^. A meta-analysis evaluated various US-based malignancy risk stratification systems (such as the Korean Thyroid Imaging Reporting and Data System (K-TIRADS), and the American College of Radiology Thyroid Imaging Reporting and Data System (ACR-TIRADS)) etc. to determine their effectiveness in distinguishing FTC from FTA^7^. The study found that these systems had limited diagnostic performance, with area under the curve (AUC) values ranging from 0.511 to 0.611, indicating insufficient sensitivity and specificity for reliable differentiation between the two conditions^7^. The Chinese Thyroid Imaging Reporting and Data System (C-TIRADS) system showed the highest AUC of 0.611 but still demonstrated low sensitivity (26.9%) and moderate specificity (95.4%) for FTC detection^7^. Another study^8^ identified specific US features associated with malignancy in follicular lesions. Key features included tumor protrusion, microcalcifications, irregular margins, marked hypoechogenicity, and irregular shape, which were significantly correlated with FTC risk. However, these characteristics were not universally effective in distinguishing FTC from FTA due to their overlap in presentation^8,9^. The difficulty in distinguishing between FTC and FTA highlights the need for more advanced diagnostic techniques. Accurate differentiation of these tumors is essential for determining the most effective management and treatment strategies^10,11^. To enhance the objectivity and accuracy of thyroid nodule assessment, numerous researchers have started developing Computer-Aided Diagnosis models. These models are designed to extract features from US images, aiding clinicians in making more precise and unbiased evaluations^12–15^.

Deep neural networks, particularly pre-trained convolutional neural networks (CNNs), have emerged as powerful tools in the classification of thyroid nodules, significantly enhancing diagnostic accuracy and efficiency in medical imaging^16–18^. The application of these advanced models addresses several challenges inherent in traditional diagnostic methods.

Pre-trained CNNs benefit from pre-processing techniques that normalize images and remove artifacts, ensuring that variations in texture and scale do not hinder the learning process^19,20^. This calibration allows CNNs to focus on relevant features of the nodules^19,20^.

This study aims to use transfer learning to train a deep CNN specifically, models such as MobileNetV2, VGG16, ResNet50, ResNet152, and ResNet101, all pretrained on the ImageNet dataset for the analysis of thyroid US images to distinguish between FTC and FTA. The anticipated findings are intended to give physicians significant insights, improving their capacity to differentiate between FTC and FTA, which is critical for making prompt and suitable treatment decisions. This study further highlights the importance of continuous research in thyroid pathology and imaging, with the ultimate goal of enhancing diagnostic techniques and patient outcomes in thyroid tumor care.

## Materials and methods

### Patients

**Fig. 1:**
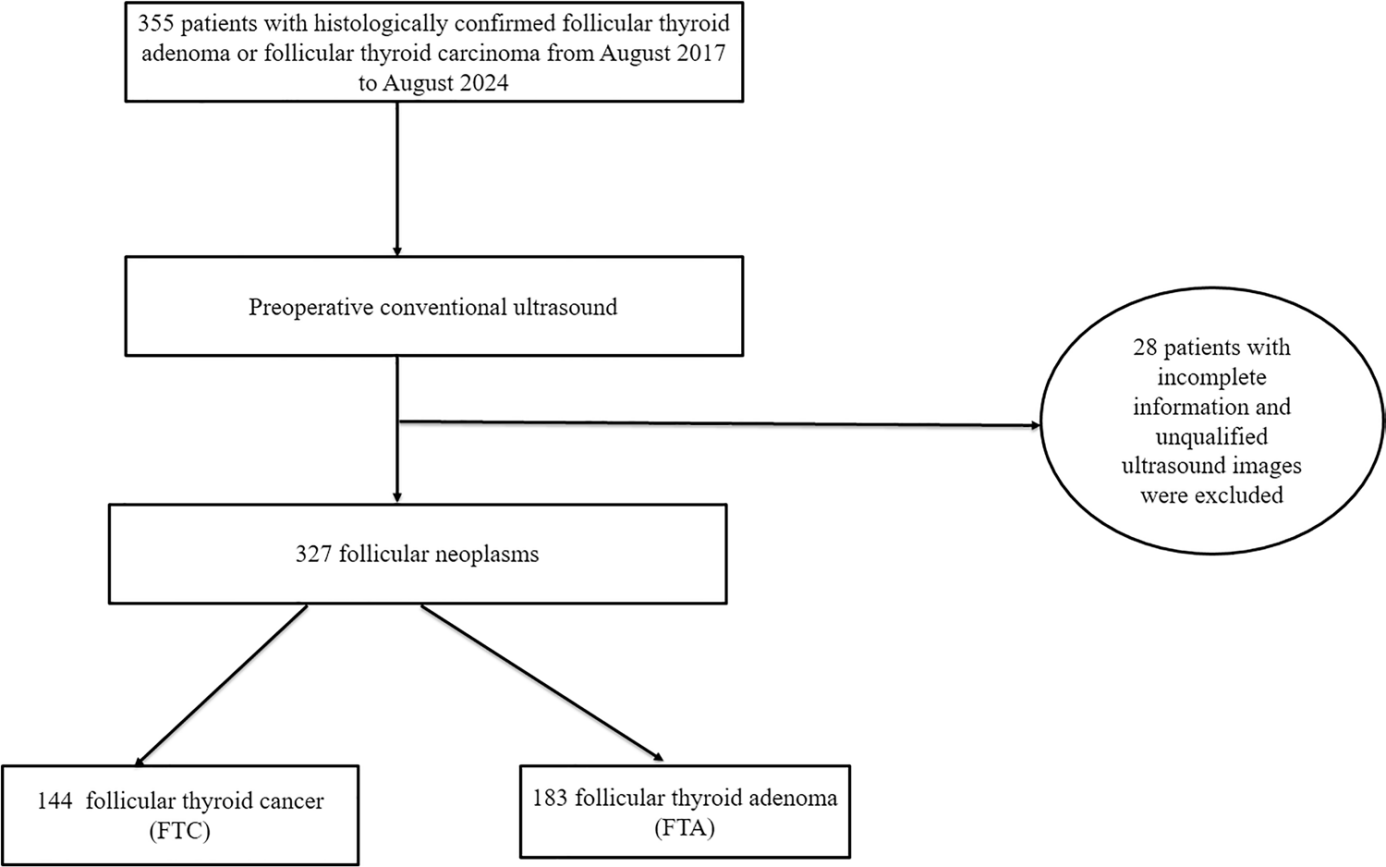
Schematic diagram of patient selection. US, ultrasound.

This study was approved by the Ethics Committee of the Affiliated Hospital of Integrated Traditional Chinese and Western Medicine and the requirement for informed consent was waived. FTC patients who underwent a preoperative thyroid US examination and histologically confirmed diagnosis of FTC at Affiliated Hospital of Integrated Traditional Chinese and Western Medicine between August 2017 and August 2024, were retrospectively enrolled. Furthermore, patients with histologically confirmed FTA between August 2017 and August 2024 were also enrolled. Figure 1 shows the enrolment procedure. The inclusion criteria were having undergone preoperative grayscale US examination of the thyroid, with related US images and diagnostic results obtained; a maximum nodule diameter > 1 cm, < 5 cm; histopathological evaluation-confirmed FTC or FTA; and unilateral and single focal lesion. The exclusion criteria were unclear US images of nodules owing to artifacts and a maximum nodule diameter < 1 cm.

This study included 327 patients, each presenting with a single thyroid nodule. The cohort comprised 261 women (79.82%) and 66 men (20.18%), with a mean age of 47.07 ± 14.44 years. Among the 327 follicular neoplasms, histopathological evaluation of surgical specimens identified 183 cases (55.96%) as FTAs (mean age, 48.89 ± 12.96 years) and 144 cases (44.04%) as FTCs (mean age, 44.77 ± 15.89 years). Using stratified sampling, patients were randomly assigned to a training cohort (*n* = 263) and a test cohort (*n* = 64) in an 8:2 ratio.

## Ultrasound examination and image pre-processing

Before surgery, all patients underwent a routine US examination conducted by experienced sonographers with over 10 years of experience in performing thyroid US examinations. The imaging was performed using a Hi Vision Preirus (Hitachi Healthcare), Aloka ProSound F75 (Hitachi Healthcare), EPIQ7 (Philips Healthcare, Eindhoven, Netherlands), IU22 (Philips Healthcare, Eindhoven, Netherlands), LOGIQ E9 (GE Medical Systems, USA), Acuson S3000 (Siemens Healthineers), Acuson Sequaia (Siemens Healthineers), US system, equipped with a 5–12 MHz linear array transducer. The thyroid and cervical areas were examined using both longitudinal and transverse continuous scanning techniques. This enabled the assessment of various tumor characteristics, including maximum diameter, composition, margin, halo, echo texture, internal septations, nodule within the nodule, and calcifications.

Retrospective analysis of US features was used to classify each thyroid nodule according to two stratification systems: ACR-TIRADS and C-TIRADS^21,22^. For statistical analysis, nodules were dichotomized into two groups: a positive predictive group for FTC (categories 4 and 5 in ACR-TIRADS; categories 4B to 5 in C-TIRADS) and a negative predictive group for FTC (categories 1 to 3 in ACR-TIRADS; categories 2 to 4 A in C-TIRADS).

The region of interest (ROI) of the primary thyroid lesion for each US image was segmented by a sonographer with over 10 years of thyroid US examination experience using the GIMP software (https://www.gimp.org/). Based on the ROI of each lesion, the top, bottom, left, and right boundary points were automatically generated to create the bounding box. The rectangular bounding box was then cropped from the original image.

To eliminate noise from the images, a discrete wavelet transform (DWT)-based denoising approach was used. The method uses wavelets’ multi-resolution capabilities to extract noise (high-frequency components) from essential visual characteristics (low-frequency components). First, the grayscale US images were divided into approximation and detail coefficients using DWT. The high-frequency detail coefficients, which are largely noisy, were thresholded using the Median Absolute Deviation (MAD) approach, with soft thresholding used to suppress the noise. The threshold was established adaptively using the noise level of the wavelet coefficients. Following thresholding, the image was reconstructed with the inverse DWT, which combined the remaining low-frequency and thresholded high-frequency components to produce a denoised image. The images were resized to 224 × 224 pixels, normalized, and fed into the CNNs as an input layer.

## Construction of the models

In this study, VGG16, ResNet101, MobileNetV2, ResNet152, and ResNet50 were employed as the base models for training. Transfer learning was used to fine-tune the model’s weights and biases, significantly reducing training time. Besides, pretrained parameters from the ImageNet dataset were initially loaded, and then our dataset was used to retrain the model. To adapt the model for our binary classification task, the original ImageNet classifier was replaced with a binary classifier, producing a class probability vector between 0 and 1 as the prediction for each patient. The model was trained from scratch using a cross-entropy loss function and the Adam optimizer, with a learning rate of 0.0001 and a batch size of 32. Given the limited training data available in this study and the need to mitigate overfitting and sample imbalances, a method called data augmentation was employed^23,24^, which meant randomly horizontal and vertical flipping the input image, randomly adjusting the height of an image by an amount of 0.2, randomly adjusting the width of an image by an amount of 0.2, randomly zooming into an image by an amount of 0.2, randomly rotating an image by an amount of 0.2. This process ensures that the model focuses on identifying thyroid lesions amidst potential noise sources^24^. The study further utilized pre-trained models to leverage learned features from balanced datasets, which enhances model performance on our datasets. Herein, in the evaluation of the models, the study included metrics that are better suited for imbalanced datasets, such as Area under the Receiver Operating Characteristic Curve (AUC-ROC), rather than only accuracy. PyTorch 2.2.2 and Keras version 2.10.0 was used to implement the training and testing codes utilizing Python (version 3.10.12). The models underwent 20 epochs of training to prevent overfitting.

## Model performance

ROC curve analysis along with the area under the curve (AUC) and its 95% confidence interval (CI) were used to interpret the discriminatory performance of the models. Confusion matrices were employed to visualize model performance, while loss and accuracy curves were analyzed to assess training dynamics. Additionally, key performance metrics including accuracy, sensitivity, specificity, F1 score, positive predictive value (PPV), and negative predictive value (NPV), all with 95% CIs—were computed. To further evaluate the clinical utility and calibration of each model, decision curve analysis (DCA) and calibration curve analysis were also conducted.

### Statistical analysis

IBM SPSS Statistics for Windows version 26.0 (Armonk, New York, USA) and Python 3.10.12 were used for the statistical analysis. Pearson’s chi-square test or Fisher’s exact test was used to compare categorical characteristics. The independent sample t-test was used for continuous variables with a normal distribution, whereas the Mann-Whitney U test was used for those without. A two-sided P value of < 0.05 indicated a statistically significant difference.

## Results

### Clinical characteristics

This study included 327 patients, each presenting with a single thyroid nodule. The cohort comprised 261 women (79.82%) and 66 men (20.18%), with a mean age of 47.07 ± 14.44 years. Among the 327 follicular neoplasms, histopathological evaluation of surgical specimens identified 183 cases (55.96%) as FTAs and 144 cases (44.04%) as FTCs. The most common US characteristics of follicular neoplasms in this cohort were solid composition (91.74%), isoechoic echogenicity (88.07%), regular margins (70.95%), calcifications (87.16%), heterogeneous texture (61.77%), absence of a halo (84.10%), lack of internal septations (97.24%), and no evidence of a “nodule within a nodule” (99.08%) (Table 1).

**Table 1:** Participant baseline characteristics.

Variable		Category/Group		Count		Percentage (%)		Mean ± SD
Age								47.07 ± 14.44
Sex		Female		261.0		79.82		
		Male		66.0		20.18		
Diameter		> 4 cm		151.0		46.18		
		> 3 cm		77.0		23.55		
		> 2 cm		60.0		18.35		
		< 2 cm		39.0		11.93		
Composition		Cystic		25.0		7.65		
		Solid		300.0		91.74		
		Predominantly solid		2.0		0.61		
Margin		Regular		232.0		70.95		
		Irregular		95.0		29.05		
Halo		Absent		275.0		84.10		
		Irregular halo		52.0		15.90		
Echo		Anechoic		3.0		0.92		
		Isoechoic		288.0		88.07		
		Very hypoechoic		36.0		11.01		
Texture		Even		125.0		38.23		
		Uneven		202.0		61.77		
Internal septations		Absent		317.0		96.94		
		Present		10.0		3.06		
Nodule within nodule		Absent		324.0		99.08		
		Present		3.0		0.92		
Calcifications		Absent		285.0		87.16		
		Present		42.0		12.84		
ACR-TIRADS		ACR-TR1		0		0.00		
		ACR-TR2		63.0		19.27		
		ACR-TR3		107.0		32.72		
		ACR-TR4		157.0		48.01		
		ACR-TR5		0.00		0.00		
C-TIRADS		C-TR2		0.00		0.00		
		C-TR3		314.0		96.02		
		C-TR4A C-TR4B to 5		13.0 0.00		3.98 0.00		

TI-RADS, Thyroid Imaging Reporting and Data System; ACR, American College of. Radiology; C–TI-RADS, Chinese TI-RADS; SD, standard deviation.

**Table 2:** Participant characteristics of the FTA and FTC groups.

Variable		Category/Group		FTA		FTC		*P*-value	
Age				48.89 ± 12.96		44.77 ± 15.89		0.01	
Sex		Female		150 (81.97%)		111 (77.08%)		0.34	
		Male		33 (18.03%)		33 (22.92%)			
Diameter		< 2 cm		22 (12.02%)		17 (11.81%)		0.55	
		> 2 cm		34 (18.58%)		26 (18.06%)			
		> 3 cm		48 (26.23%)		29 (20.14%)			
		> 4 cm		79 (43.17%)		72 (50.00%)			
Composition		Cystic		23 (12.57%)		2 (1.39%)		0.00	
		Solid		159 (86.89%)		141 (97.92%)			
		Predominantly solid		1 (0.55%)		1 (0.69%)			
Margin		Regular		171 (93.44%)		61 (42.36%)		0.00	
		Irregular		12 (6.56%)		83 (57.64%)			
Halo		Absent		174 (95.08%)		101 (70.14%)		0.00	
		Irregular halo		9 (4.92%)		43 (29.86%)			
Echo		Anechoic		3 (1.64%)		0 (0.00%)		0.00	
		Isoechoic		169 (92.35%)		119 (82.64%)			
		Very hypoechoic		11 (6.01%)		25 (17.36%)			
Texture		Even		105 (57.38%)		20 (13.89%)		0.00	
		Uneven		78 (42.62%)		124 (86.11%)			
Internal septations		Absent		183 (100.00%)		134 (93.06%)		0.00	
		Present		0 (0.00%)		10 (6.94%)			
Nodule within nodule		Absent		183 (100%)		141 (97.92%)		0.17	
		Present		0 (0.00%)		3 (2.08%)			
Calcifications		Absent		172 (93.99%)		113 (78.47%)		0.00	
		Present		11 (6.01%)		31 (21.52%)			
ACR-TIRADS		ACR-TR1		0 (0.00%)		0 (0.00%)			
		ACR-TR2		56 (30.60%)		7 (4.86%)		0.00	
		ACR-TR3		79 (43.17%)		28 (19.44%)			
		ACR-TR4		48 (26.23%)		109 (75.7%)			
		ACR-TR5		0 (0.00%)		O (0.00%)			
C-TIRADS		C-TR2		0 (0.00%)		0 (0.00%)			
		C-TR3		182 (99.45%)		132 (91.67%)		0.00	
		C-TR4A		1 (0.55%)		12 (8.33%)			
		C-TR4B to 5		0 (0.00%)		0 (0.00%)			

TI-RADS, Thyroid Imaging Reporting and Data System; ACR, American College of.

TI-RADS, Thyroid Imaging Reporting and Data System; ACR, American College of Radiology; C–TI-RADS, Chinese TI-RADS; SD, standard deviation; FTA, follicular thyroid adenoma; FTC, follicular thyroid cancer.

No significant differences were observed between FTA and FTC in terms of sex, maximum nodule diameter, or the presence of a “nodule within a nodule” (*p* > 0.05). However, significant differences were noted for the following US features: composition, margins, halo, echogenicity, texture, internal septations, and calcifications (*p* < 0.05 for all; Table 2). Additionally, age (*p* < 0.05), C-TIRADS (*p* < 0.05), and ACR-TIRADS (*p* < 0.05) scores demonstrated significant differences between the two groups (Table 2).

## Diagnostic performance of the models

**Fig. 2:**
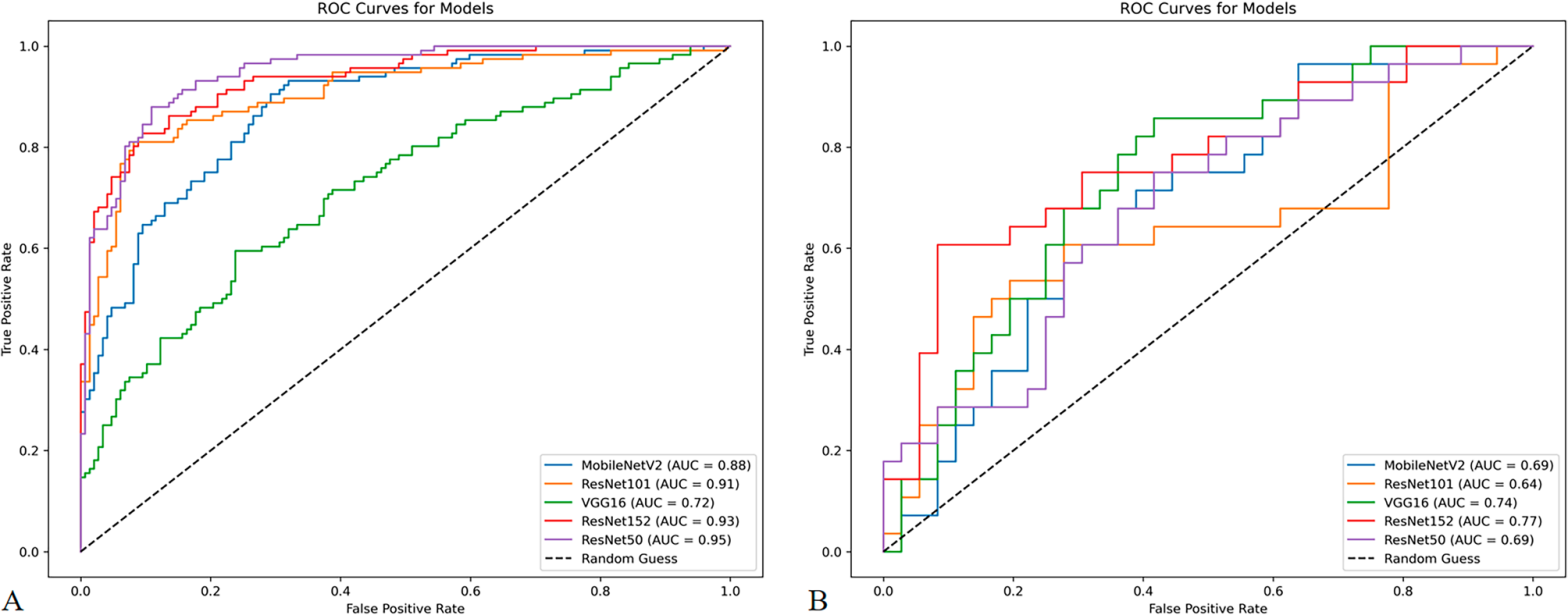
ROC curves of the models. ROC, receiver operating characteristic. (A) Training cohort (B) Test cohort.

**Fig. 3:**
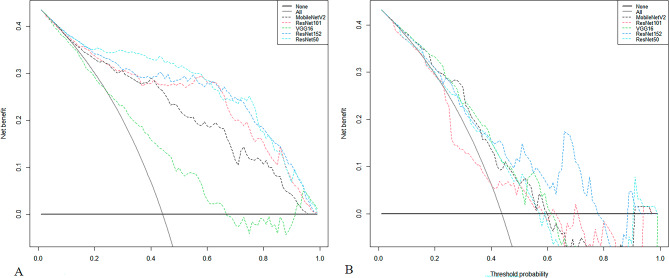
Decision curve analysis (A) Training (B) Test.

**Fig. 4:**
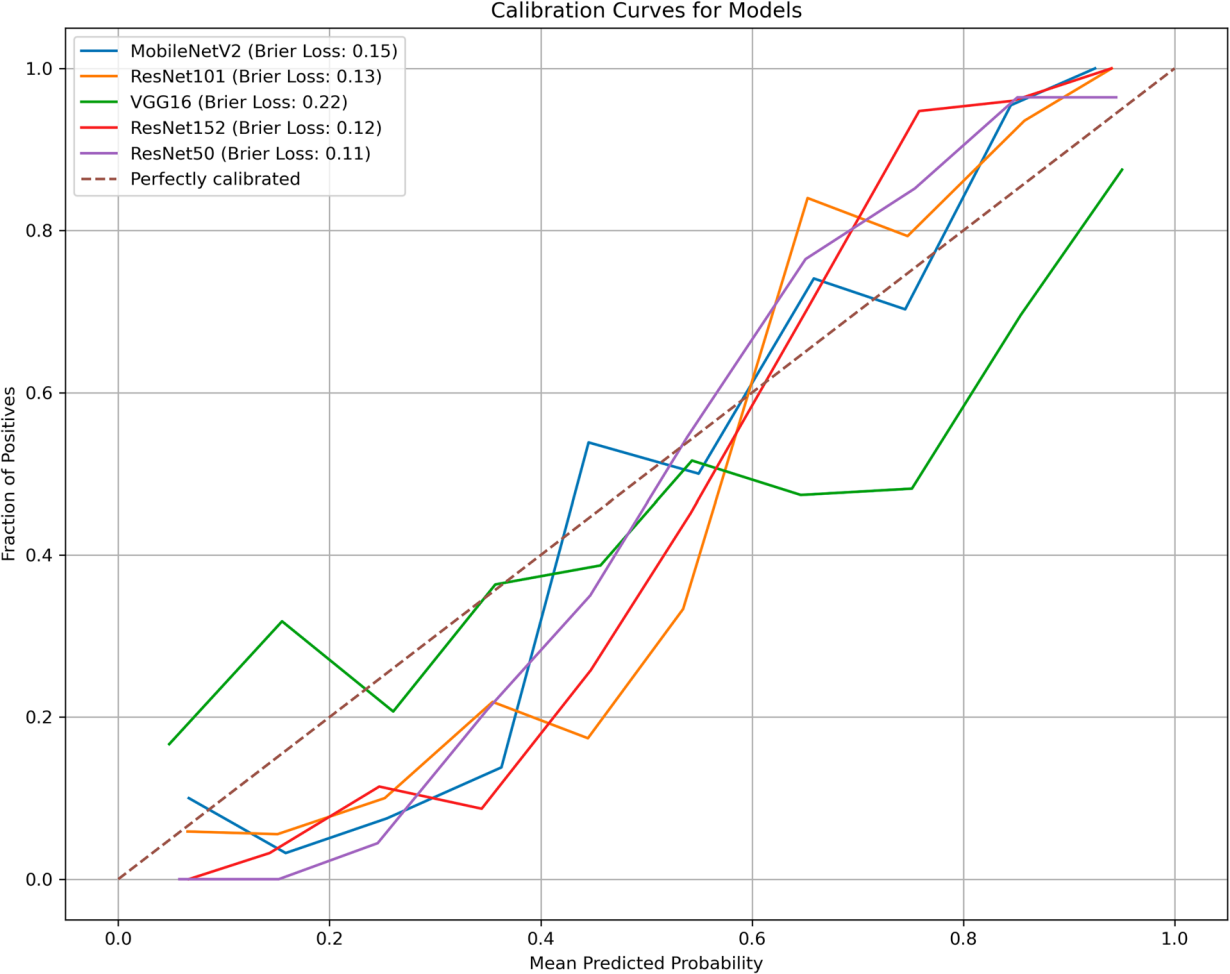
Calibration curves of the models.

The diagnostic performance of the five models based on predictive classifications is summarized in Table 3. In the test cohort, the AUC values for the five models ranged from 0.64 to 0.77. The ResNet152 model demonstrated the highest AUC (0.77; 95% CI, 0.67–0.87) for distinguishing between FTC and FTA. The corresponding diagnostic indices for the ResNet152 model were as follows: accuracy of 0.67(95% CI, 0.56–0.79), sensitivity of 0.75 (95% CI, 0.64–0.86), specificity of 0.61 (95% CI, 0.44–0.78), PPV of 0.60 (95% CI, 0.43–0.76), NPV of 0.76 (95% CI, 0.59–0.92), and F1 score of 0.76 (95% CI, 0.59–0.92). The ROC curves for all models are depicted in Fig. 2. To assess the clinical utility of the models, DCA was performed, calculating the net benefits at varying threshold probabilities in both the training and test cohorts. DCA results indicated that all models outperformed the all-or-no treatment strategy, as shown in Fig. 3. The calibration curves demonstrated moderate calibration of the models, as illustrated in Fig. 4. Furthermore, we employed the confusion matrix to assess the models’ performance. The confusion matrices depicted in Supplementary Figs. 1 and 2 quantitatively represent the model’s classification performance on the training (Supplementary Fig. 1) and test (Supplementary Fig. 2) cohorts. They also provide insight into the model’s behavior when separating true positives, false positives, true negatives, and false negatives. These matrices offer crucial details on the models’ predictive abilities.

**Table 3:** Performance summary of different models for prediction of FTC.

Model	Accuracy	AUC	Sensitivity	Specificity	F1	PPV	NPV
Test cohort							
MobileNetV2	0.63 (0.51–0.74)	0.69 (0.57–0.8)	0.71 (0.6–0.82)	0.56 (0.39–0.75)	0.63 (0.51–0.74)	0.56 (0.39–0.71)	0.71 (0.52–0.87)
ResNet101	0.62 (0.51–0.74)	0.64 (0.52–0.75)	0.61 (0.49–0.73)	0.64 (0.49–0.79)	0.59 (0.47–0.71)	0.57 (0.39–0.75)	0.68 (0.51–0.83)
VGG16	0.63 (0.51–0.74)	0.74 (0.63–0.85)	0.89 (0.82–0.97)	0.41 (0.26–0.58)	0.68 (0.56–0.79)	0.54 (0.40–0.69)	0.83 (0.63-1.00)
ResNet152	0.67 (0.56–0.79)	0.77 (0.67–0.87)	0.75 (0.64–0.86)	0.61 (0.44–0.78)	0.67 (0.55–0.78)	0.6 (0.43–0.76)	0.76 (0.59–0.92)
ResNet50	0.63 (0.51–0.74)	0.69 (0.57–0.8)	0.75 (0.64–0.86)	0.53 (0.37–0.68)	0.64 (0.52–0.75)	0.55 (0.39–0.71)	0.73 (0.57–0.88)
Train cohort							
MobileNetV2	0.78 (0.73–0.83)	0.88 (0.84–0.92)	0.8 (0.75–0.85)	0.77 (0.70–0.83)	0.77 (0.71–0.82)	0.73 (0.65–0.81)	0.83 (0.77–0.89)
ResNet101	0.84 (0.79–0.88)	0.91 (0.87–0.94)	0.85 (0.81–0.9)	0.82 (0.76–0.89)	0.82 (0.78–0.87)	0.79 (0.72-86)	0.88 (0.82–0.93)
VGG16	0.66 (0.6–0.72)	0.72 (0.66–0.77)	0.65 (0.59–0.7)	0.67 (0.59–0.74)	0.63 (0.57–0.68)	0.6 (0.51–0.69)	0.71 (0.63–0.78)
ResNet152	0.84 (0.8–0.89)	0.93 (0.9–0.96)	0.87 (0.83–0.91)	0.82 (0.76–0.87)	0.83 (0.79–0.88)	0.8 (0.73–0.86)	0.89 (0.84–0.94)
ResNet50	0.87 (0.83–0.91)	0.95 (0.92–0.97)	0.88 (0.84–0.92)	0.86 (0.80–0.92)	0.86 (0.81–0.9)	0.84 (0.77–0.90)	0.9 (0.85–0.95)

In deep learning, the loss function plays a critical role in guiding model training. Its primary objective is to minimize the loss value, indicating improved model performance. If the loss fluctuates instead of continuously decreasing, it may suggest that the model is not learning effectively. Furthermore, if the loss decreases on the training set but remains stagnant on the validation or test set, this could be indicative of overfitting. In the present study, categorical cross-entropy was employed to compute the loss for the models. As shown in Supplementary Fig. 3 both the training and validation or test data demonstrated a decrease in loss, reflecting the models’ strong performance.

Additionally, an analysis comparing C-TIRADS and ACR-TIRADS systems for differentiating FTC from FTA was conducted. The diagnostic indices for both systems, based on predictive classifications, are summarized in Table 4. The AUC values for these two systems ranged from 0.50 to 0.61. Among the two, the ACR-TIRADS system achieved the highest AUC for distinguishing FTC from FTA (0.61; 95% CI, 0.57–0.64), with the following diagnostic indices: sensitivity of 0.23 (95% CI, 0.16–0.30), specificity of 0.98 (95% CI, 0.96–1.00), positive predictive value (PPV) of 0.92 (95% CI, 0.81–1.00), negative predictive value (NPV) of 0.62 (95% CI, 0.57–0.67), and F1 score of 0.37 (95% CI, 0.27–0.45). When comparing the performance of the developed models with that of the C-TIRADS and ACR-TIRADS systems, the models developed in this study demonstrated superior performance.

**Table 4:** Performance summary of ACR-TIRADS and C-TIRADS.

METRICS	ACR-TIRADS	C-TIRADS
Accuracy	0.65 (CI: 0.60–0.70)	0.56 (CI: 0.51–0.61)
AUC	0.61 (CI: 0.57–0.64)	0.50 (CI: 0.50–0.50)
Sensitivity	0.23 (CI: 0.16–0.30)	0.00 (CI: 0.00–0.00)
Specificity	0.98 (CI: 0.96–1.00)	1.00 (CI: 1.00–1.00)
F1 Score	0.37 (CI: 0.27–0.45)	0.00 (CI: 0.00–0.00)
PPV	0.92 (CI: 0.81–1.00)	0.00 (CI: 0.00–0.00)
NPV	0.62 (CI: 0.57–0.67)	0.56 (CI: 0.51–0.61)

AUC, area under the curve; NPV, negative predictive value; PPV, positive predictive value; TI-RADS, Thyroid Imaging Reporting and Data System; ACR, American College of Radiology; C–TI-RADS, Chinese TI-RADS.

### Interpretability of the model

**Fig. 5:**
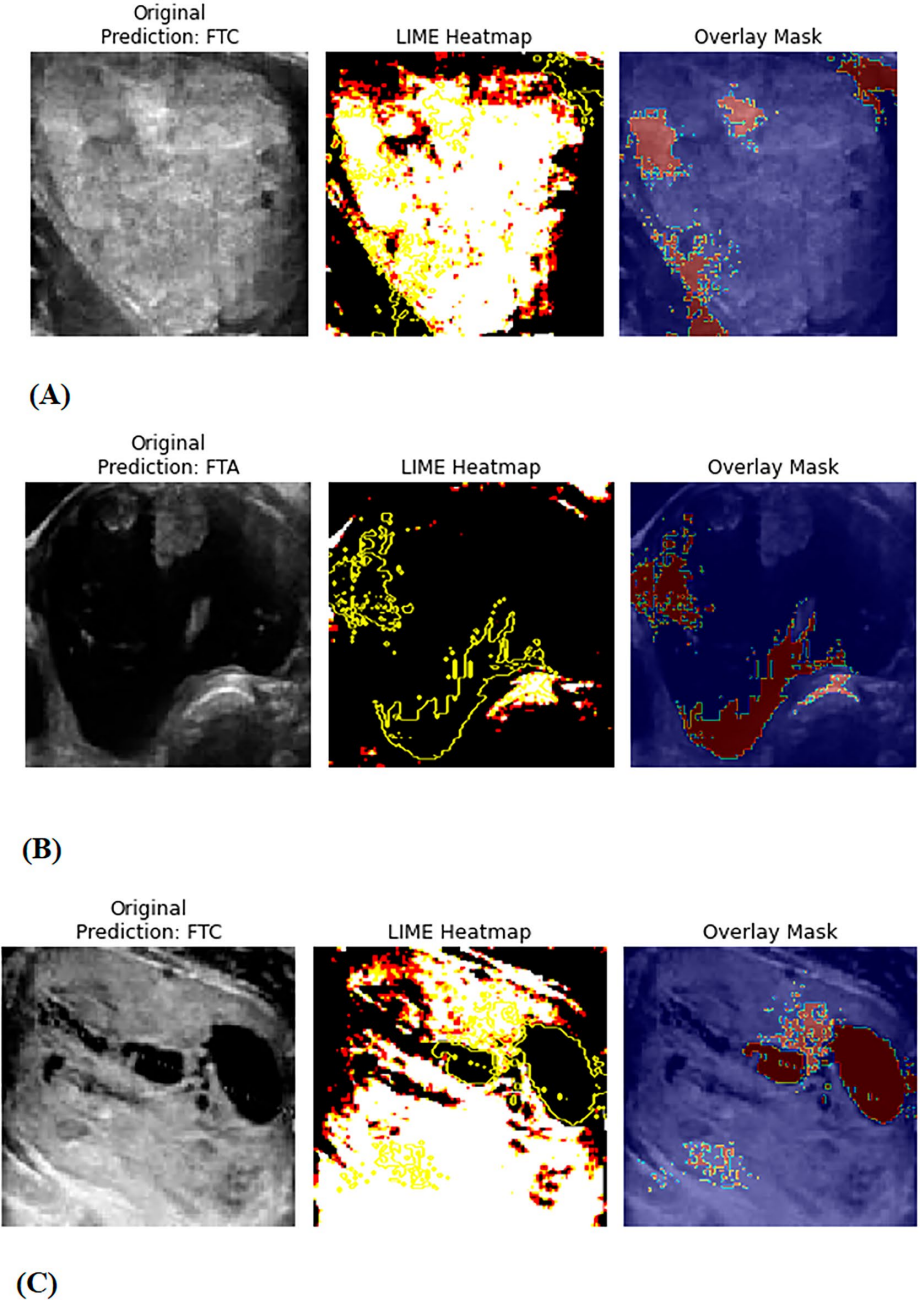
LIME (Local Interpretable Model-agnostic Explanations) visualization of the ResNet152 model’s decision-making process on three sample ultrasound images of thyroid nodules. Each row (A–C) represents one sample. The first column displays the original grayscale ultrasound images. The second column presents LIME heatmaps highlighting the most influential regions contributing to the model’s prediction. Brighter areas indicate stronger contributions. The third column shows the overlay of the LIME mask (in red) on the original image, marking the regions the model focused on.

Figure 5 shows the Local Interpretable Model-agnostic Explanations (LIME) explanation results, which provide vital insights into the best-performing model’s interpretability and decision-making process for classifying FTC and FTA using US images. The highlighted regions in the three images differ depending on the predicted lesion type, indicating that the model has acquired various discriminative features. In FTC cases (Figs. 5A and C), the LIME heatmaps mostly focus on the nodule’s interior areas. This implies that the model relies on intra-nodular traits such as internal echogenicity, hypoechoic regions, and heterogeneity features often linked with malignancy in thyroid nodules^25,26^.

This internal focus is consistent with clinical observations in which varied internal echoes and irregular hypoechoic patterns are indicative of malignancy.

In contrast, in the FTA scenario (Fig. 5B), LIME focuses on the nodule’s border regions. This attention pattern indicates that the model views edge regularity and smooth margins as significant elements in benign classification. This pattern is congruent with clinical experience, where well-defined and smooth nodule edges are often indicators of benignity^27^. The LIME mask overlay indicates that the model focuses on clinically relevant parts of the US images while ignoring irrelevant background structures or noise. This alignment of the model’s attention to established sonographic features improves the model’s interpretability and trustworthiness, which is critical for clinical acceptability. Explainable AI (XAI) approaches, like as LIME, are thus useful in evaluating model predictions, especially in sensitive medical imaging applications where explaining the rationale behind decisions is critical^28,29^.

## Discussion

Preoperative differentiation of FTC from FTA is an inherently challenging aspect of effective management for thyroid nodules. Previous studies have demonstrated that existing risk stratification systems exhibit poor performance, with AUC values ranging from 0.51 to 0.61, in differentiating FTC from FTA^7^. Consistent with prior studies, in this study, using high or intermediate suspicious stratification as the positive cutoff for FTC, the C-TIRADS and ACR-TIRADS classification systems’ AUC values ranged from 0.50 to 0.61, showing that they have a limited ability to reliably predict malignancy. This limited predictive capacity adds to the systems’ failure to successfully distinguish between FTC and FTA^30^. The poor performance of US-based malignancy risk stratification systems for thyroid nodules, particularly in distinguishing FTC from FTA, can be attributed to several factors, including overlapping US features. Follicular neoplasms frequently present with similar US characteristics, making it difficult for classification systems to differentiate between FTC and FTA accurately. This overlap may lead to misclassification and poor diagnostic performance^8^. These findings underscore the need to develop more accurate and advanced diagnostic systems for evaluating and classifying follicular thyroid neoplasms.

Five CNN models to distinguish FTC from FTA using US images were developed and evaluated in this study. Three key findings emerged from our analysis. First, all five CNN models demonstrated the ability to differentiate FTC from FTA, achieving AUC values ranging from 0.64 to 0.77. Specifically, MobileNetV2, ResNet101, VGG16, ResNet152, and ResNet50 achieved AUCs of 0.69, 0.64, 0.74, 0.77, and 0.69, respectively. Second, among the evaluated CNN architectures, ResNet152 exhibited the highest predictive performance. These findings highlight the potential of deep learning-based approaches to improve the accuracy of FTC versus FTA classification in clinical practice. Third, the CNN models outperformed conventional stratification systems, including the C-TIRADS and the ACR-TIRADS. The superior performance of the CNN models developed in this study, compared to conventional stratification systems, can be attributed to the exceptional ability of CNNs to detect and analyze complex spatial patterns and subtle textural variations within imaging data. This capability is particularly critical for distinguishing morphologically similar lesions, such as FTC and FTA, where visual differences may be subtle and challenging to discern using traditional approaches. Traditional stratification systems may rely on simpler criteria that do not capture the nuanced differences present in US images, leading to less accurate classifications.

Using a CNN with 8-bit bitmap US images, Seo et al. successfully distinguished between FTC and FTA, obtaining an impressive AUC of 0.809^31^. In another study, Shin et al. employed artificial neural networks (ANN) and support vector machines (SVM) based on preoperative ultrasonography data to address the same classification task. However, their models demonstrated comparatively lower performance, with accuracies of 0.741 and 0.69, respectively^32^. Our best-performing CNN model, ResNet-152, achieved a competitive AUC of 0.77, demonstrating its promise as a dependable diagnostic tool for preoperative assessment. While our model’s performance is slightly lower than Seo et al.‘s results, the findings demonstrate the feasibility of using deep learning architectures to help distinguish FTC from FTA and emphasize the need for further optimization and validation in larger, multi-center datasets.

Supplementary Fig. 3 illustrates the loss curves demonstrate a consistent downward trend, reflecting the iterative refinement of the model’s parameters and the effective minimization of the error function. This highlights model’s capacity to extract salient features from US images, enabling precise classification and robust generalization to unseen data. The models were trained for 20 epochs, with each epoch requiring between 44 and 58 s on a CPU, for a total training period of about 15 to 20 min. It had an average inference time of 125 milliseconds per image on a CPU and a total evaluation time of 8 s for two batches of test data. These findings indicate that the model may give near-real-time predictions, making it potentially useful for clinical applications. However, additional optimization, like as GPU acceleration or quantization, may improve inference speed for real-time deployment.

Besides, DCA was employed to evaluate the clinical utility of the CNN models. The results, as illustrated in Fig. 3, demonstrated that the CNN models yielded a higher overall net benefit compared to both the treat-all and treat-none strategies. This finding suggests that the proposed CNN models can substantially enhance clinical decision-making for FTC prevention. Notably, the models outperformed standard care, particularly at moderate threshold probabilities, highlighting their potential to improve patient outcomes by accurately identifying individuals at risk who may benefit from early intervention.

Calibration is crucial because it ensures that the risk predictions made by a model are reliable. A well-calibrated model helps healthcare professionals make informed decisions and provides patients with accurate information about their health risks. As shown in Fig. 4, while the calibration of the models was not perfect, the ResNet152 and ResNet50 models displayed relatively higher calibration performance compared to the other models. This shows that ResNet152 and ResNet50 may produce more trustworthy probability estimates in this setting.

Furthermore, in this study most of the model’s misclassification errors occurred when FTC and FTA had overlapping US features, making differentiation difficult even for experienced radiologists. Specifically, several false negatives involved FTC cases that lacked typical malignant features, whereas false positives were benign FTA cases with suspicious characteristics such as irregular margins or heterogeneous echotexture.

Despite their extensive use, existing TI-RADS-based risk stratification methods have limited ability to properly characterize follicular-patterned lesions^7^. These diagnostic problems are aggravated by the high rate of inconclusive FNA cytology results, which frequently necessitate diagnostic lobectomy for a definitive diagnosis^25,33^. The current CNN-based model overcomes these constraints by providing an automated, non-invasive adjunct that improves diagnosis accuracy while potentially minimizing unnecessary surgeries.

With real-time predictive capabilities and a significant reduction in interobserver variability in US interpretation, CNN models can provide clear benefits in thyroid nodule assessment, adding to mounting evidence that deep learning models can outperform radiologists in nodule classification, especially through improved false-positive/negative rates^13,14,34–36^. Combining AI-driven classifications with conventional US descriptors may improve diagnostic accuracy and aid in medical decision-making^14,37^.

Through the use of LIME, which finds critical picture regions and improves interpretability, the CNN models used in this study produce clinically useful outputs such as classification probabilities and explainable features^38^. Integration could be used in practice as an adjuvant for indeterminate FNA results or as a triage tool for high-risk nodules. To achieve smooth workflow integration and proper physician oversight, successful clinical adoption will include cooperative improvement with radiologists, endocrinologists, and pathologists^39^.

While our CNN models produced encouraging results, a few limitations need to be addressed in future studies. The small sample size and single-center data may limit generalizability by failing to adequately reflect a broad population and potentially introducing biases related to demographics, imaging procedures, and clinical care. Furthermore, the retrospective nature of the TIRADS assessment may result in inconsistencies due to operator-dependent differences in US acquisition and interpretation. The lack of external validation reduces the model’s applicability in real-world scenarios. To address these issues, the research group intends to conduct a prospective, multicenter study with a bigger, more diverse dataset, standardized imaging techniques, and multi-operator validation to enhance model resilience and generalizability.

## Conclusion

The findings of this study demonstrated that CNN models performed satisfactorily in distinguishing FTC from FTA in patients with follicular neoplasms. Among the evaluated CNN architectures, ResNet152 exhibited the highest predictive performance. The CNN models outperformed conventional stratification systems, including the C-TIRADS and the ACR-TIRADS. These findings highlight the potential of deep learning-based approaches to improve the accuracy of FTC versus FTA classification in clinical practice.

## Electronic supplementary material

Below is the link to the electronic supplementary material.

Supplementary Material 1

## Data Availability

The data that support the findings of this study are available from the corresponding author upon reasonable request.

## Abbreviations

ATA-TIRADS: American thyroid association thyroid imaging reporting and data system
ACC: Accuracy
ACR -TIRADS: American College of radiology thyroid imaging reporting and data system
ANN: Artificial neural networks
AUC: Area under the receiver operating characteristic curve
CI: Confidence interval
CNN: Convolutional neural network
C-TIRADS: Chinese thyroid imaging reporting and data system
DCA: Decision curve analysis
DWT: Discrete wavelet transform
FTA: Follicular thyroid adenoma
FTC: Follicular thyroid carcinoma
K-TIRADS: Korean thyroid imaging reporting and data system
MAD: Median absolute deviation
NPV: Negative predictive value
PPV: Positive predictive value
PTC: Papillary thyroid carcinoma
ROC: Receiver operating characteristic
ROI: Region of interest
SVM: Support vector machine
US: Ultrasound

## Declarations

### Declaration of AI-assisted Technologies

During the preparation of this manuscript, the authors have utilized the basic feature of the OpenAI’s GPT model, solely for the purpose of eliminating grammatical errors and improving the overall readability of the document. The authors have thoroughly reviewed the final manuscript and take full responsibility for the content of the published article.
