# Supplementary material for: Ultrasound-based classification of follicular thyroid Cancer using deep convolutional neural networks with transfer learning
Source: Sci Rep. 2025 Jul 1;15:21708. doi: 10.1038/s41598-025-05551-7 (PMC12216321; doi:10.1038/s41598-025-05551-7)
Supplement: Supplementary file 1 — Supplementary Material 1 [file 41598_2025_5551_MOESM1_ESM.docx]

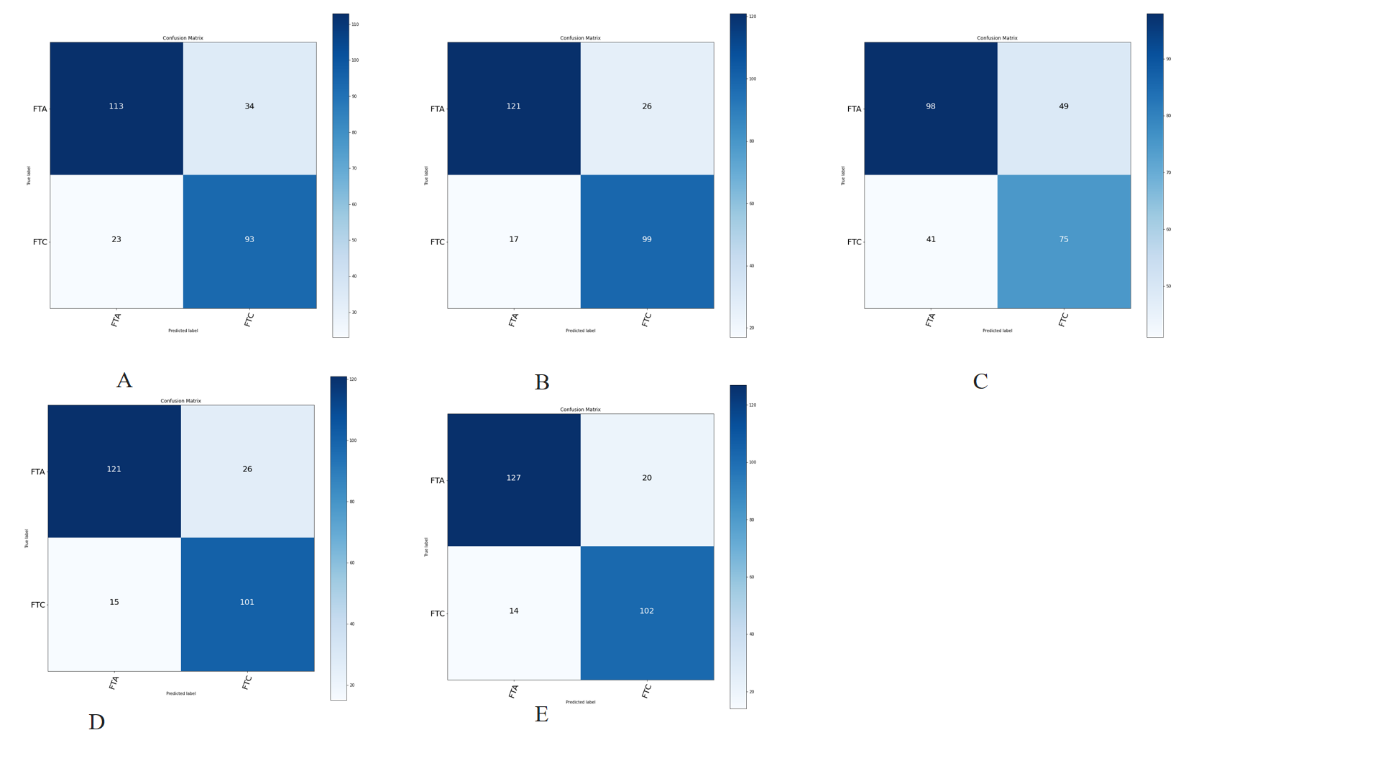


Supplementary Figure 1: Confusion matrix. The 2 × 2 contingency table reports the number of true positives, false positives, false negatives, and true negatives


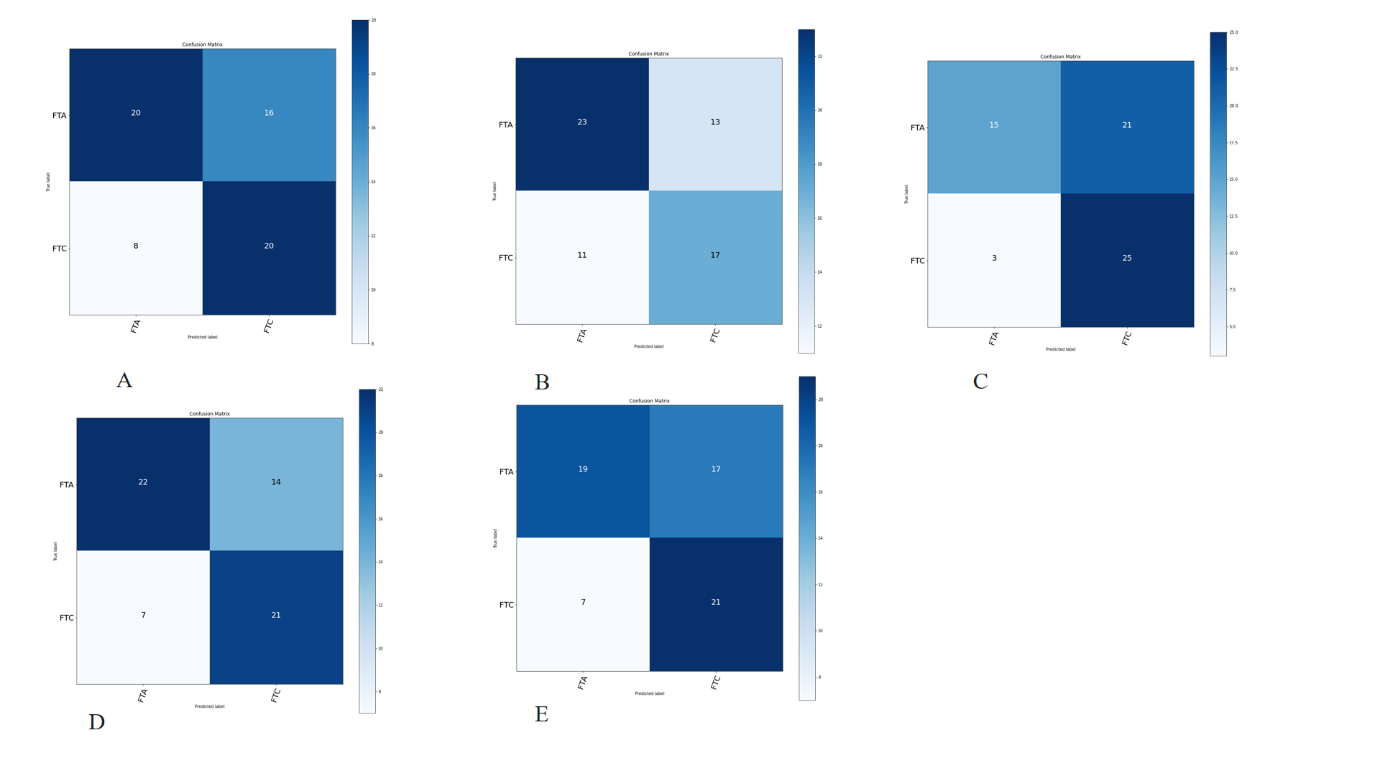


Supplementary Figure 2: Confusion matrix. The 2 × 2 contingency table reports the number of true positives, false positives, false negatives, and true negatives


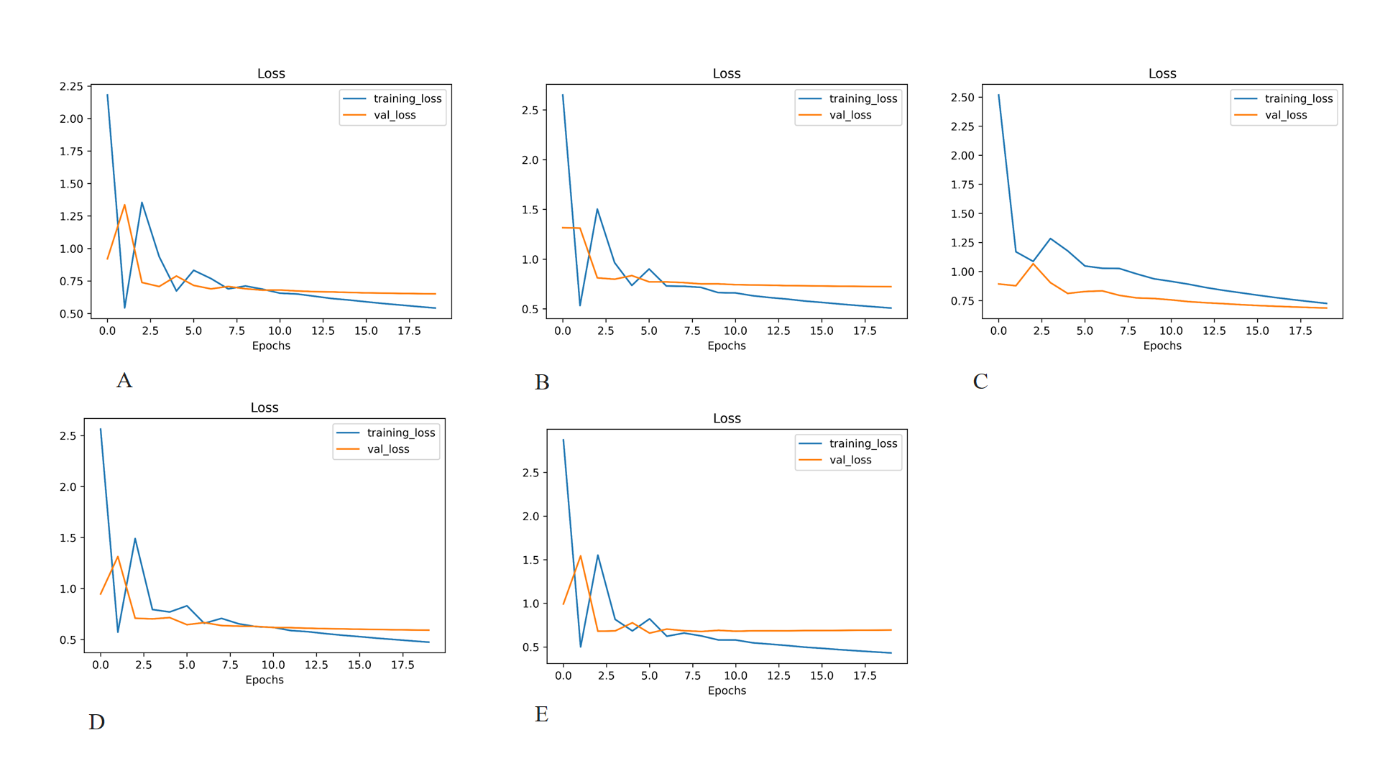


Supplementary Figure 3: Loss curves of the models. Blue and yellow curves represent the training and validation or test data, respectively. (A) MobileNetV2 (B) ResNet101 (C) VGG16 (D) ResNet152 (E) ResNet50
